# Epoxygenated Fatty Acids Inhibit Retinal Vascular Inflammation

**DOI:** 10.1038/srep39211

**Published:** 2016-12-14

**Authors:** Megan E. Capozzi, Sandra S. Hammer, Gary W. McCollum, John S. Penn

**Affiliations:** 1 Vanderbilt University, Department of Molecular Physiology and Biophysics, Nashville, TN, USA; 2 Vanderbilt University Medical Center, Department of Ophthalmology and Visual Sciences, Nashville, TN, USA

## Abstract

The objective of the present study was to assess the effect of elevating epoxygenated fatty acids on retinal vascular inflammation. To stimulate inflammation we utilized TNFα, a potent pro-inflammatory mediator that is elevated in the serum and vitreous of diabetic patients. In TNFα-stimulated primary human retinal microvascular endothelial cells, total levels of epoxyeicosatrienoic acids (EETs), but not epoxydocosapentaenoic acids (EDPs), were significantly decreased. Exogenous addition of 11,12-EET or 19,20-EDP when combined with 12-(3-adamantane-1-yl-ureido)-dodecanoic acid (AUDA), an inhibitor of epoxide hydrolysis, inhibited VCAM-1 and ICAM-1 expression and protein levels; conversely the diol product of 19,20-EDP hydrolysis, 19,20-DHDP, induced *VCAM1* and *ICAM1* expression. 11,12-EET and 19,20-EDP also inhibited leukocyte adherence to human retinal microvascular endothelial cell monolayers and leukostasis in an acute mouse model of retinal inflammation. Our results indicate that this inhibition may be mediated through an indirect effect on NFκB activation. This is the first study demonstrating a direct comparison of EET and EDP on vascular inflammatory endpoints, and we have confirmed a comparable efficacy from each isomer, suggesting a similar mechanism of action. Taken together, these data establish that epoxygenated fatty acid elevation will inhibit early pathology related to TNFα-induced inflammation in retinal vascular diseases.

One third of the estimated 285 million diabetics world-wide show signs of diabetic retinopathy (DR), and one third of these have progressed to vision threatening DR^1^. DR presents in two clinically distinct forms commonly referred to as non-proliferative (NPDR) and proliferative (PDR) diabetic retinopathy, corresponding to the early and late stages of DR progression, respectively. NPDR is characterized by fundus abnormalities including microaneurysms, hemorrhages and vasoregression whereas PDR is defined by the presence of preretinal neovascularization^2^,^3^,^4^. Inflammatory cytokines are elevated early in DR pathogenesis and presumably contribute to advancement of DR to later disease stages. Tumor necrosis factor-α (TNFα) is a potent pro-inflammatory cytokine, and its elevated serum and vitreous levels in DR patients correlate with disease progression and morbidity^5^,^6^,^7^,^8^. Furthermore, deletion of TNFα inhibited diabetes-induced leukostasis and retinal vascular leakage in mice^9^. TNFα induces the expression of the leukocyte adhesion proteins vascular cell adhesion molecule-1 (*VCAM1*) and intercellular adhesion molecule-1 (*ICAM1*) in human retinal endothelial cells^10^,^11^. Both function in the firm adherence of leukocytes facilitating their immobilization; a process referred to as leukostasis. When leukocytes adhere to the luminal surface of the retinal capillary endothelium, they are believed to cause the formation of vaso-occlusive thrombi that can obstruct blood flow, causing oxygen starvation and subsequent capillary death. The death of retinal capillaries is a pivotal point in DR pathogenesis because it results in the formation of focal areas of retinal ischemic that become hypoxic. In response to hypoxia, the retina elaborates vascular endothelial cell grow factor (VEGF) and other growth factors that promote the onset of PDR along with its associated vision-threatening morbidities. Adherent leukocytes also secrete noxious stimuli, such as reactive oxygen species and inflammatory cytokines, which further contribute to inflammation, blood-retina barrier breakdown, retinal vasoregression, and edema; all hallmarks of DR^12^,^13^,^14^,^15^. Interestingly, studies have shown that genetic deletion or antibody blockade of ICAM-1 has beneficial effects on multiple pathogenic outcomes in experimental DR, including leukostasis and vascular permeability^15^,^16^.

Diabetes is known to alter multiple pathways involved in endogenous fatty acid metabolism. Tissues can metabolize fatty acids to biologically active lipid mediators through the cyclooxygenase (COX), lipoxygenase (LOX) or cytochrome P450 epoxygenase (CYP) pathways. While COX, LOX, and their products have received considerable attention in the diabetic retina^17^,^18^,^19^,^20^,^21^,^22^,^23^,^24^,^25^, relatively little is known about the role of retinal CYP-derived lipid mediators. CYP enzymes are endoplasmic reticulum membrane-bound monooxygenases that oxidize a variety of substrates, including polyunsaturated fatty acids. Two of these, arachidonic acid (AA) and docosahexaenoic acid (DHA), are found in relatively high abundance in the retinal vasculature^26^, thus their metabolites may be of particular importance to retinal vascular homeostasis. A subset of CYP enzymes, including the well-characterized CYP2C8, CYP2C9 and CYP2J2 in humans, epoxygenate AA and DHA to produce epoxyeicosatrienoic acids (EET) and epoxydocosapentaenoic acids (EDP), respectively. CYP enzymatic activity yields four EET (5,6-EET, 8,9-EET, 11,12-EET, 14,15-EET) and five EDP (7,8-EDP, 10,11-EDP, 13,14-EDP, 16,17-EDP, 19,20-EDP) regioisomers. These epoxides, and particularly the EET products, are known to exert a number of potent anti-inflammatory activities in diverse vasculature beds, including reduced *VCAM1* and *ICAM1* expression^27^,^28^,^29^,^30^. Soluble epoxide hydrolase (sEH) hydrolyzes EET and EDP to less biologically active diols (dihydroxyeicosatrienoic acid (DHET) and dihydroxydocosapentaenoic acid (DHDP), respectively)^31^,^32^, thereby reducing EET and EDP tissue levels and consequently, reducing their anti-inflammatory effects. Accordingly, sEH represents a rational and promising therapeutic target. Thus, its inhibition has been the objective of multiple clinical trials in diabetes-related pathology^32^,^33^,^34^,^35^,^36^. More specifically, these clinical trials have been directed at the treatment of hypertension and glucose tolerance in pre-diabetic patients, and pulmonary disease in obese male smokers^33^,^34^. Notably, sEH inhibition has not yet been examined as a treatment for retinal disease.

Reduced levels of EET are observed in the vitreous of patients with NPDR and PDR^5^, and the decreased epoxide levels may be permissive for increased retinal inflammation. Accordingly, therapeutic interventions that raise EET and EDP levels may be expected to ameliorate DR inflammation; however, these approaches have not been assessed. Based on these considerations, we hypothesized that treatment of primary human retinal microvascular endothelial cells (HRMEC) with a DR-relevant inflammatory cytokine, TNFα, would decrease epoxide production, limiting their anti-inflammatory effects. Conversely, elevation of epoxide levels by their exogenous addition as well as sEH inhibition may block cytokine-induced inflammatory events in HRMEC, providing a basis for a viable therapeutic strategy to inhibit the retinal vascular inflammation that is observed at the onset of diabetic retinopathy.

## Results

### TNFα Decreases Epoxyeicosatrienoic Acid Levels in HRMEC-Conditioned Medium

**Figure 1:**
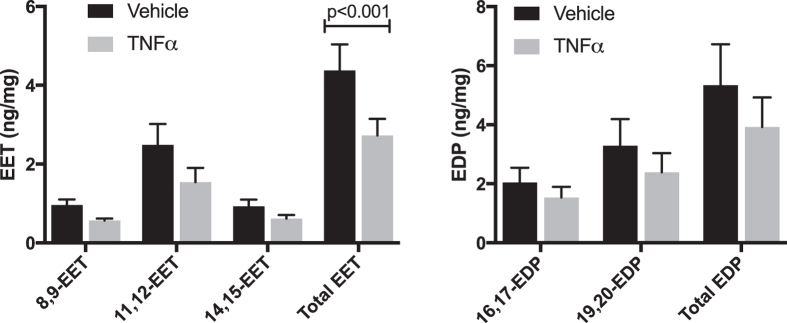
The effect of TNFα on epoxygenated fatty acid levels. HRMEC were treated with vehicle or TNFα (1 ng/ml), arachidonic acid and docosahexaenoic acid substrates were provided, and the level of their epoxygenated products was measured in the conditioned media by LC-MS/MS. These data are normalized to the total protein of the cell lysates. Each bar represents the mean ± SEM (n = 8 for EET measurements; n = 9 for EDP measurements).

TNFα has previously been shown to alter levels of the cytochrome P450 enzymes that produce epoxygenated fatty acids^37^,^38^, however any effects of TNFα on EET or EDP production by endothelial cells has not been determined. To investigate these effects, HRMEC were treated with TNFα followed by treatment with substrate (AA and DHA; 10 μM each), and epoxide levels were measured in the conditioned medium. Although no specific regioisomer was significantly decreased, the sum of all EET regioisomers was significantly reduced by 37.6% (p < 0.001). While not significant, total EDP was reduced by 26.5% (Fig. 1).

### 11,12-EET or 19,20-EDP in combination with AUDA Inhibit, and 19,20-DHDP Promotes, TNFα-induced *VCAM1* and *ICAM1* Expression

**Figure 2:**
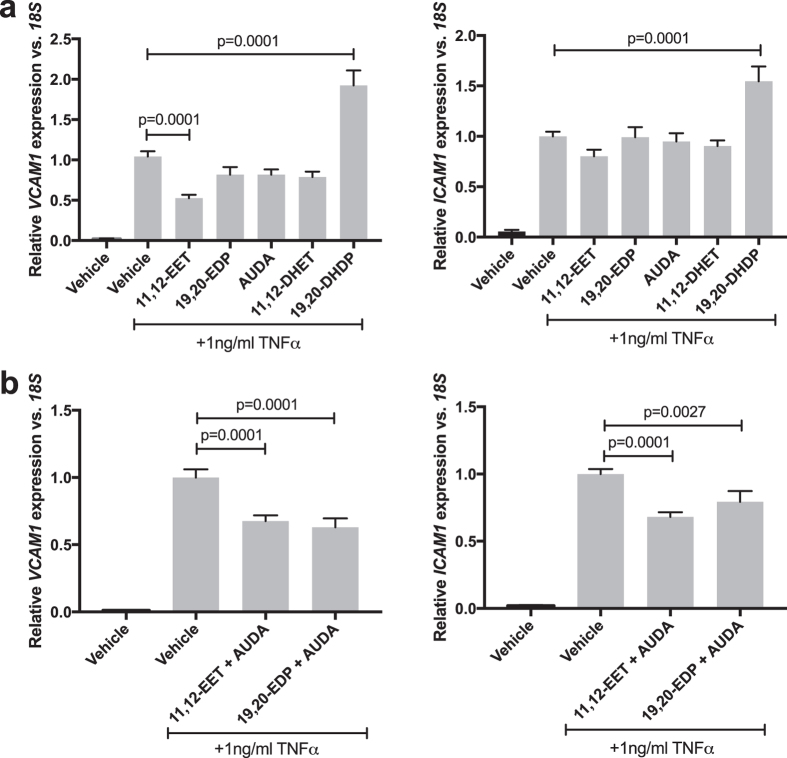
The effect of 11,12-EET, 19,20-EDP, AUDA, or the corresponding diols on TNFα-induced adhesion molecule expression. HRMEC were treated with TNFα in the presence or absence of (**a**) 11,12-EET (0.5 μM), 19,20-EDP (0.5 μM), AUDA (10 μM), 11,12-DHET (0.5 μM), or 19,20-DHDP (0.5 μM); or (**b**) combinations of 11,12-EET or 19,20-EDP with AUDA. Expression of *VCAM-1* and *ICAM-1* was assessed by qRT-PCR analysis. Each bar represents the mean ± SEM (a: n = 6; b: n = 12).

We investigated the effects of the exogenous addition of epoxides or sEH inhibition of TNFα-induced inflammation in HRMEC cultures. sEH activity was inhibited using 12-(3-adamantane-1-yl-ureido)-dodecanoic acid (AUDA). AUDA stabilizes both exogenous and endogenous EET and EDP by blocking hydrolysis of the epoxides to diols, extending their biological half-lives to presumably potentiate any biological effects. In Supplementary Figure S1, we verified that hydrolysis of epoxides was reduced in HRMEC cultures following AUDA treatment. HRMEC cultures were treated with TNFα in the presence or absence of exogenous 11,12-EET, 19,20-EDP, AUDA, or the EET and EDP diols, 11,12-DHET and 19,20-DHDP and *VCAM1* and *ICAM1* expression was assessed. The concentrations of the epoxides (0.5 μM) or the sEH inhibitor AUDA (10 μM) were determined from preliminary studies and fall within typical ranges used in the literature^27^,^39^,^40^,^41^,^42^,^43^. As shown in Fig. 2a, 11,12-EET significantly reduced (49.6%; p = 0.0001) and 19,20-DHDP increased (84.6%; p = 0.001) TNFα-induced *VCAM1* expression, while the other treatments yielded no significant effect on *VCAM1*. None of these treatments had an effect on *ICAM1* expression, with the exception of 19,20-DHDP, which increased TNFα-induced *ICAM1* expression by 54.5% (p = 0.0001).

Next, we treated TNFα-stimulated HRMEC with combinations of 11,12-EET or 19,20-EDP and AUDA and assessed any effects on *VCAM1* and *ICAM1* expression. As shown in Fig. 2b, 11,12-EET plus AUDA inhibited *VCAM1* by 32.5% (p = 0.0001) and *ICAM1* by 31.9% (p = 0.0001). Similarly, 19,20-EDP plus AUDA inhibited *VCAM1* by 37% (p = 0.0001) and *ICAM1* by 20.7% (p = 0.0027).

### 11,12-EET or 19,20-EDP with sEH Inhibition Reduces TNFα-stimulated VCAM-1 and ICAM-1 Protein Levels

**Figure 3:**
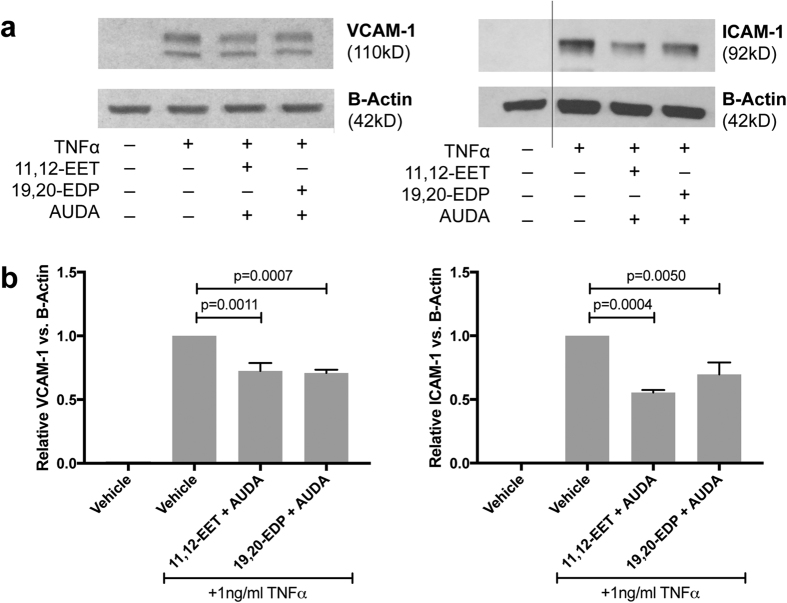
The effect of 11,12-EET or 19,20-EDP plus sEH inhibition on TNFα-induced VCAM-1 and ICAM-1 protein levels. (**a**) Representative blots from HRMEC treated with TNFα in the presence or absence of 11,12-EET (0.5 μM) or 19,20-EDP (0.5 μM) with AUDA (10 μM); and (**b**) quantification of 3 individual blots. Each bar represents the mean ± SEM (n = 3).

To further validate the effect of 11,12-EET or 19,20-EDP in combination with AUDA, VCAM-1 and ICAM-1 protein levels were assessed. HRMEC were treated with TNFα in the presence or absence of 11,12-EET or 19,20-EDP plus AUDA for 4 hours; VCAM-1 and ICAM-1 levels were determined by immunoblot analysis. As shown in Fig. 3, 11,12-EET plus AUDA inhibited both VCAM-1 and ICAM-1 levels by 27.5% (p = 0.0007) and 44.5% (p = 0.0004), respectively. 19,20-EDP plus AUDA performed similarly, inhibiting VCAM-1 by 29.2% (p = 0.0011) and ICAM-1 by 30.3% (p = 0.005).

### 11,12-EET or 19,20-EDP with sEH inhibition Reduces PBMC Adhesion to HRMEC Monolayers

**Figure 4:**
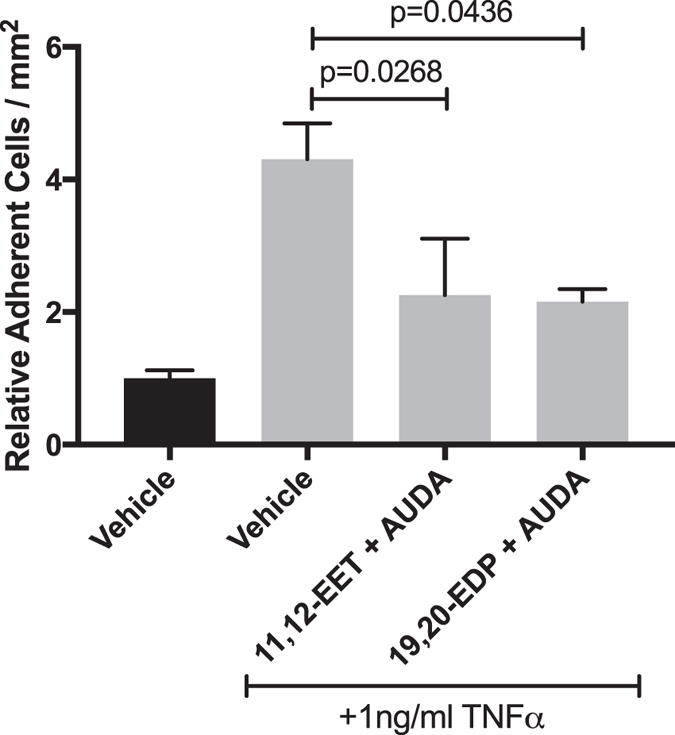
The effect of 11,12-EET or 19,20-EDP plus AUDA on TNFα-induced leukocyte adhesion to HRMEC monolayers. HRMEC monolayers were treated TNFα in the presence or absence of 11,12-EET (0.5 μM) or 19,20-EDP (0.5 μM) with AUDA (10 μM), and PBMC were then flowed over the treated monolayers in a parallel plate flow chamber. Each bar represents the mean ± SEM (vehicle: n = 8; TNFα: n = 9; 11,12-EET + AUDA: n = 6; 19,20-EDP: n = 4).

We performed leukocyte adhesion assays to determine how EET- and EDP-dependent VCAM-1 and ICAM-1 expression and protein levels affect TNFα-induced leukocyte adhesion. HRMEC monolayers were cultured in a parallel plate flow chamber and treated with TNFα in the presence or absence of 11,12-EET or 19,20-EDP plus AUDA for 4 hours. Untreated peripheral blood mononuclear cells (PBMC) were flowed over the monolayers and adhesion was measured. As shown in Fig. 4, TNFα induced PBMC adhesion by 4.3-fold; 11,12-EET or 19,20-EDP plus AUDA inhibited this induction by 47.6% (p = 0.0268) and 49.9% (p = 0.0436), respectively.

### 11,12-EET or 19,20-EDP with sEH Inhibition Reduces TNFα-induced NFκB Activity

**Figure 5:**
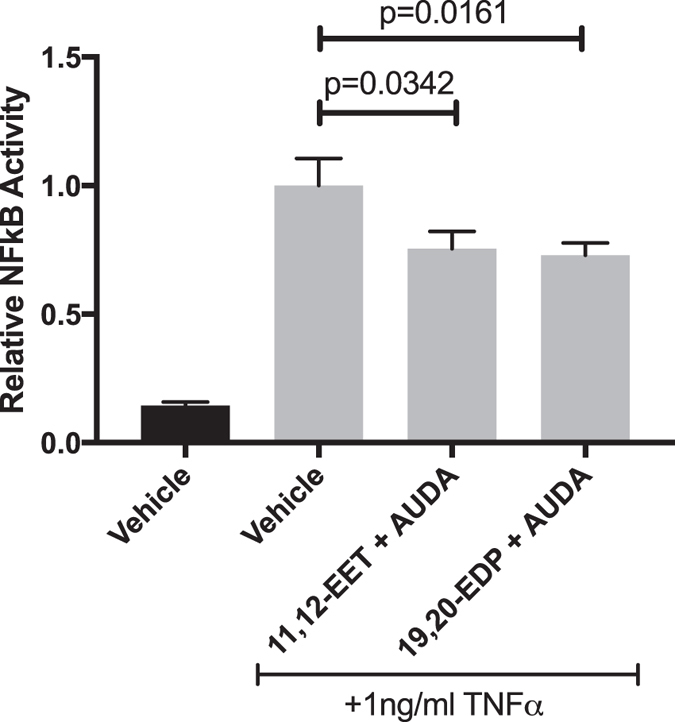
The effect of 11,12-EET or 19,20-EDP plus AUDA on TNFα-induced NFκB Activation. HRMEC were transfected with luciferase constructs and treated with TNFα in the presence or absence of 11,12-EET (0.5 μM) or 19,20-EDP (0.5 μM) with AUDA (10 μM). NFκB activity was determined by measuring the ratio of firefly-to-Renilla luciferase. Each bar represents the mean ± SEM (n = 20).

In various macrovascular endothelial cell types, 11,12-EET exerts its anti-inflammatory effects by preventing IκBα degradation and subsequent NFκB translocation^27^,^42^,^44^. To assess whether 11,12-EET and 19,20-EDP are working through this mechanism, NFκB activity was measured using a promoter assay. HRMEC were transfected with a luciferase promoter construct and treated with TNFα in the presence or absence of 11,12-EET or 19,20-EDP plus AUDA. As shown in Fig. 5, 11,12-EET or 19,20-EDP plus AUDA inhibited TNFα-induced NFκB activation by 24.6% (p = 0.0342) and 28% (p = 0.0161), respectively. However, this inhibition was not via a direct effect on IκBα degradation (Supplementary Figure S2).

### 11,12-EET or 19,20-EDP with sEH Inhibition Mitigates TNFα-induced Retinal Leukostasis

**Figure 6:**
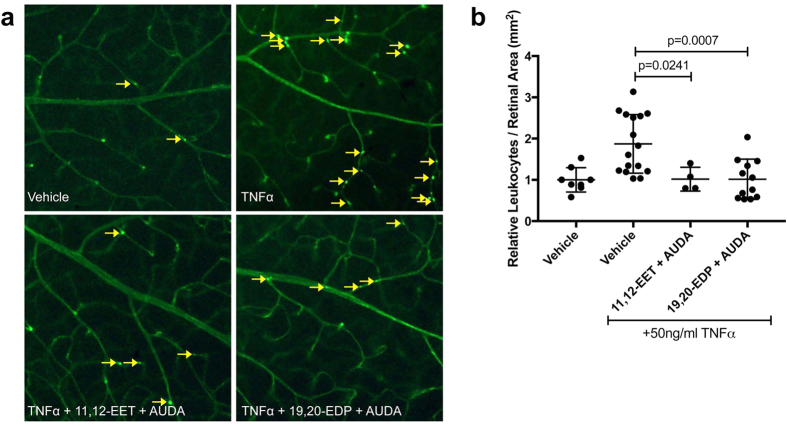
The effect of 11,12-EET or 19,20-EDP plus AUDA on TNFα-induced retinal leukostasis. Mice were injected intravitreally with 50 ng/ml TNFα in the presence or absence of 11,12-EET (0.5 μM) or 19,20-EDP (0.5 μM) with AUDA (10 μM). (**a**) Representative images of retinal flatmounts with Concanavalin-A perfusion; yellow arrows indicate adhered leukocytes; (**b**) quantification of adherent leukocytes normalized to retinal area. Bars represent mean ± SD (vehicle: n = 8; TNFα: n = 16; 11,12-EET + AUDA: n = 4; 19,20-EDP: n = 12).

For proof-of-concept *in vivo*, we assessed the effects of elevating EET or EDP levels on an acute mouse model of retinal inflammation. C57BL/6J mice received intravitreal injections of TNFα in the presence or absence of 11,12-EET or 19,20-EDP plus AUDA; adherence of leukocytes to retinal vessels was analyzed 6 hours post-injection. As shown in Fig. 6, TNFα induced retinal vascular leukocyte adherence by 87.3% (p = 0.0024), similarly to previous reports^11^. Co-treatment with either 11,12-EET or 19,20-EDP plus AUDA completely mitigated the TNFα-induced adherence of leukocytes in retinal vessels (p = 0.0241 and p = 0.0007, respectively).

## Discussion

Our data demonstrated for the first time that 11,12-EET and 19,20-EDP are similarly efficacious against TNFα-induced vascular inflammation. While EET is well established as an anti-inflammatory lipid that inhibits the induction of leukocyte adhesion molecules in various endothelial cell types^27^,^28^,^29^,^42^, these activities have never been demonstrated for EDP. Interestingly, the EET and EDP precursors, AA and DHA respectively, have opposing effects on leukocyte adhesion molecule expression in retinal endothelial cells. AA increases VCAM-1 and ICAM-1 levels^45^, while DHA decreases TNFα-induced VCAM-1 and ICAM-1 levels^46^,^47^. In the present study, we demonstrated that these opposing roles are no longer observed when using the epoxygenated products, 11,12-EET and 19,20-EDP. It is important to note that the rate of epoxide biosynthesis is limited by substrate availability^48^. Though DHA is abundant in the retina, this is almost exclusively due to enrichment in the retinal photoreceptor outer segment membranes. In the retinal vasculature, AA and DHA are found in equal amounts^26^, suggesting that both substrates, and therefore their products, would likely be found in similar levels at the site of epoxide generation in the vasculature. Thus, both AA- and DHA-derived epoxides may contribute similarly to retinal vascular homeostasis, particularly relating to inflammation.

In this study, we used only one of the four EET regioisomers (11,12-EET) and one of the five EDP regioisomers (19,20-EDP). 11,12-EET was used due to its high relative abundance in retinal endothelial cell cultures (Fig. 1) and retinal tissue^49^, as well as its proven anti-inflammatory capacity compared to other regioisomers^27^,^50^. These data suggest that changes in the levels of 11,12-EET may be highly biologically relevant in the retina. Similarly, 19,20-EDP is the most abundant DHA-derived epoxide product in the retina^49^,^51^,^52^. Also, 19,20-EDP is the least efficiently metabolized sEH substrate of the DHA-derived epoxides, suggesting that therapeutic levels may be more easily achieved compared to the other regioisomers^51^.

While diol products are often inactive in the vasculature, in the retina, the 19,20-DHDP has been shown to promote developmental angiogenesis^49^. In the present study, we demonstrate a pro-inflammatory activity for this diol product in retinal endothelial cells. This finding further argues for the use of sEH inhibition as an efficacious therapeutic strategy because it would not only promote elevated levels of anti-inflammatory epoxygenated fatty acids, but also reduce the production of their pro-inflammatory diol products.

The sEH enzyme is highly expressed in the endothelium throughout a variety of tissue beds^39^,^53^,^54^,^55^,^56^, and we demonstrated its activity in HRMEC cultures (Supplementary Figure 1). However, in the developing mouse retina, sEH immunoreactivity did not co-localize with the vascular lectin stain, isolectin GS-IB4 from *Griffonia simplicifolia*^49^. Soluble epoxide hydrolase is constitutively expressed and inducible by systemic factors. In diabetes and obesity, which is associated with a chronic systemic inflammation, sEH levels are elevated, and their levels are responsive to insulin therapy^57^,^58^. Furthermore, endothelial cell activation with homocysteine, a systemic factor associated with diabetic retinopathy^59^, induced sEH expression and protein levels as well as VCAM-1 expression, while sEH inhibition reduced homocysteine-induced VCAM-1 induction^60^. Another diabetes-relevant stimulus, angiotensin II, induced sEH levels via c-Jun binding to SP-1 sites in the 5’-flanking region^55^,^61^. TNFα has been shown to activate c-Jun in endothelial cells^62^, yet its direct effect on sEH expression has not been assessed. Thus, TNFα-induced inflammation may alter EET/EDP levels in our HRMEC cultures via increased sEH expression and activity.

While AUDA potently inhibits sEH activity, as demonstrated in Supplementary Figure 1, it is important to note that it can have off-target effects related to the end points examined in this study. For instance, Fang *et al*.^63^ demonstrated weak PPARα agonism with AUDA treatment^63^. However, in the present study AUDA does not recapitulate PPARα agonism, because PPARα activation is efficacious against TNFα-induced VCAM-1 expression^64^, but AUDA alone did not exhibit any effect on this endpoint (Fig. 2). Therefore, in the context of this study, we believe that AUDA is functioning by sEH inhibition, because it is only efficacious when paired with exogenous epoxides.

TNFα-induced retinal leukostasis was adapted for use in this study as a model of low-grade chronic inflammation related to DR, as reported previously^11^. We chose to administer a dose of TNFα (50 pg) to the vitreous cavity that was based on preliminary dose response profiles within ranges that reflect low-grade retinal inflammation similar to that observed in vitreous from patients with DR^5^,^65^. Moreover, our reported induction of retinal leukostasis is comparable to that observed in rodent models of diabetes^9^,^66^,^67^. The TNFα concentration we chose produced a significant increase in adherent retinal leukocytes with sufficient magnitude to test the addition of exogenous EET and EDP to achieve statistical significance in this acute model, while at the same time maintaining relevance to the low-grade state of inflammation observed in DR.

In our *in vitro* flow chamber experiments, we observed a partial inhibition of TNFα-induced leukocyte adherence compared to the complete mitigation of TNFα-induced leukostasis we observed in the acute mouse model of retinal inflammation. A number of reasons could explain the differences between these results. First, we narrowed our survey of inflammation-related molecular targets to VCAM-1 and ICAM-1 because they have been shown to mediate TNFα-induced leukostasis^67^,^68^,^69^, and have precedence as EET-dependent targets^27^,^28^,^29^,^30^. However, other targets are likely to contribute to leukocyte adherence in the *in vivo* setting, such as E-selectin or P-selectin, and their levels may also be EET- and/or EDP-dependent. Furthermore, though EET and EDP were locally administered, they may exert additional anti-inflammatory actions on the circulating leukocytes. Our *in vitro* experiments focused on the effects of EET and EDP on the retinal endothelial cells alone. Indeed, evidence from other studies suggests that these epoxides may also exert their effects on macrophages and monocytes^42^,^50^,^70^,^71^,^72^,^73^. Hence, EET- and EDP-dependent effects on circulating cells may represent an important component of the *in vivo* pathogenesis that is absent in our *in vitro* studies. Hence, experiments designed to test EET- and EDP-dependent effects on circulating cells in the context of retinal leukostasis are currently ongoing in our laboratory. Lastly, our *in vitro* experiments were performed in human-derived microvascular endothelial cells, and our *in vivo* endpoints were performed in mice. The published IC50 value for AUDA related the to inhibition of mouse sEH (18 nM) is lower than that reported for the human variant (69 nM)^74^. Therefore, AUDA is more potent for the mouse enzyme, and this may contribute to the improved efficacy observed in our mouse model.

This is the first study to directly compare the effects of EET and EDP on molecular and cellular events related to retinal vascular inflammation occurring in DR. While EDP has some bioactivities similar to those of EET, and in some cases demonstrating greater potencies such as in vasodilation^75^, opposite biological outcomes have also been observed. For example, EDP has been reported to be anti-angiogenic whereas EET is likely to be pro-angiogenic depending on the specific experimental context^40^,^43^,^76^,^77^. In the present study we showed a similar potency for EET and EDP to reduce *in vitro* leukocyte adhesion and leukostasis in the mouse. This suggests that the mechanisms by which they exert their anti-inflammatory effects are similar. The mechanistic details of EET and EDP bioactivities remain undetermined. A specific receptor for epoxygenated fatty acids is yet to be identified, although they do signal through a number of pathways, including PPARs and GPCRs^30^,^78^,^79^,^80^. The majority of evidence supporting EET anti-inflammatory activity points to signaling pathways that converge on the transcription factor NFκB. Node *et al*. originally showed that 11,12-EET exerted anti-inflammatory effects in bovine aortic endothelial cells by inhibiting IKK activity and IκBα degradation, thereby preventing NFκB translocation and initiation of pro-inflammatory mediator transcription^27^. While subsequent studies validated this finding, they were all performed in macrovascular endothelial cell types^42^,^44^. To our knowledge, this is the first study to show that NFκB activation is inhibited by 11,12-EET or 19,20-EDP in microvascular endothelial cells, but not via IκBα degradation. Future studies will seek to identify novel signaling mechanisms by which these epoxides exert their anti-inflammatory effects on microvascular endothelial cells as opposed to those identified in various macrovascular endothelial cells.

Taken together, our data shows for the first time that 11,12-EET and 19,20-EDP, when combined with application of the sEH inhibitor AUDA, are similarly efficacious against TNFα-induced leukocyte adherence *in vitro* and leukostasis *in vivo*. Both of these epoxides act in part by inhibiting the induction of VCAM-1 and ICAM-1. Based on the results of these studies, the addition of exogenous EET or EDP, along with their stabilization via sEH inhibitors, may represent a viable treatment strategy for DR. We believe that blocking retinal inflammation in the early stages of NPDR with EET and EDP therapy may prevent transition to PDR and its associated morbidities, including blindness. Future studies will be designed to optimize strategies focused on increasing concentrations of EET or EDP by chronic systemic administration in experimental models of DR to assess their effects over longer time courses of pathogenesis.

## Methods

### Human Retinal Microvascular Endothelial Cell Culture

Primary human retinal microvascular endothelial cells (HRMEC; Cell Systems, Kirkland, WA) were validated by assessing cytoplasmic VWF and uptake of Di-I-Ac-LDL. Cells were cultured in phenol red-free endothelial basal medium (EBM; Lonza Walkersville, MD) containing 10% FBS with SingleQuots (Lonza) and grown on attachment factor- (Cell Signaling, Danvers, MA) coated culture dishes. Cultures were incubated at 37 °C, 5% CO_2_, and 20.9% O_2_ and 95% relative humidity. Passages 6 to 8 were used for all experiments, and all data is derived from at least 3 independent experiments.

### Epoxygenated Fatty Acid Quantification

HRMEC were grown to 85% confluence, and then placed in serum-reduced (2% FBS) culture medium for 12 hours. HRMEC were treated with 1 ng/ml TNFα (Sigma-Aldrich, St. Louis, MO) or vehicle (0.1% BSA in water) for 9 hours followed by treatment with EET and EDP substrate (10 μM AA and 10 μM DHA; Sigma-Aldrich) for 3 hours. Media was collected, triphenylphosphine (TPP; Sigma-Aldrich) was added, and samples were evacuated with argon. After adding synthetic [^2^H_11_]-labeled 8,9-DHET, 11,12-DHET, 14,15-DHET, 16,17-DHDP, and 19,20-DHDP (5 ng each) as internal standards, the EET/EDPs and DHET/DHDPs were extracted with acidified CHCl_3_/CH_3_OH (2:1) and purified by silica solid phase extraction, separating EET/EDPs and DHET/DHDPs. The EET/EDPs were converted to the corresponding DHET/DHDPs by treatment with acetic acid overnight. The samples were quantified by LC/MS/MS using an Acquity BEH C18 columns (1.0 × 100 mm; 1.7 μm) connected to a TSQ-Quantum Vantage triple quadrupole spectrometer (Thermo Fisher Scientific; Waltham, MA). DHET/DHDP positional isomers were resolved using a linear solvent gradient that went from 70% 15 mM aqueous ammonium acetate (pH 8.5), 30% acetonitrile to 40% 15 mM aqueous ammonium acetate (pH 8.5), 60% acetonitrile in 6 minutes and at a flow of 0.18 ml/min. For analysis, we utilized collision-induced fragmentation of the DHET/DHDPs at m/z 337 and the [^2^H_11_]-DHET/DHDP internal standards at m/z 448. These same product ions were also used for the deuterated internal standards. Quantifications were done using the ratio of the area of the DHET/DHDP peaks compared to the area of the corresponding deuterated DHET/DHDP peaks. Cell lysates were collected, total protein was measured using a Pierce bicinchoninic acid assay (BCA; Thermo Fisher Scientific), and EET and EDP levels were normalized to the total protein.

### Quantitative Real-Time RT-PCR of *VCAM1* and *ICAM1*

HRMEC were seeded in 6-well plates and at 85% confluence, cells were placed in serum-reduced culture medium for 12 hours. Cells were treated for 2 hours with vehicle or 1 ng/ml TNFα in the presence or absence of the following treatments, either alone or in combination: 11,12-EET (0.5 μM; Sigma-Aldrich), 19,20-EDP (0.5 μM; Cayman Chemical; Ann Arbor, MI), AUDA (10 μM; Sigma-Aldrich); 11,12-DHET (0.5 μM; Cayman Chemical), or 19,20-DHDP (0.5 μM; Cayman Chemical). Cells were then washed twice with cold PBS, and total RNA was collected using an RNeasy Mini kit (Qiagen, Valencia, CA), according to the manufacturers protocol. Total RNA was reverse transcribed using the High-Capacity cDNA Archive Kit (Applied Biosystems, Waltham, MA). Quantitative RT-PCR was performed using TaqMan Gene Expression Assays (Applied Biosystems) in duplicate by co-amplification of human *VCAM1* and *ICAM1* cDNAs compared with *18S* as a normalization control.

### Immunoblot Analysis

HRMEC were seeded in 10 cm^2^ dishes and were placed in serum-reduced medium at 85% confluence. Cells were then treated with vehicle or 1 ng/ml TNFα in the presence or absence of 11,12-EET (0.5 μM) or 19,20-EDP (0.5 μM) with AUDA (10 μM) for 4 hours. Cells were then washed twice in cold PBS and lysed using radio-immunoprecipitation assay (RIPA) buffer (Qiagen) with protease inhibitors (Roche; Basel, Switzerland). Samples were equilibrated for total protein concentration using a Pierce BCA assay, subjected to 10% SDS-PAGE, and gels were transferred to nitrocellulose membranes using the iBlot system (Thermo Fisher Scientific). Membranes were blocked and probed in 5% BSA for CD54/ICAM-1 (1:1000; Cell Signaling) and β-Actin (1:4000; Thermo Fisher Scientific) or 5% milk for VCAM-1 (1:1000; Abcam; Cambrdige, UK). Blots were then labeled with horseradish peroxidase-conjugated secondary antibodies (1:2000). β-Actin was used as a loading control. Membranes were incubated in Pierce ECL Western blotting substrate and developed with a ChemiDoc MP (Bio-Rad; Hercules, CA). Blots were then quantified using ImageJ software.

### Parallel Plate Flow Chamber

HRMEC were plated on glass slides coated with attachment factor. Once confluent monolayers formed, cells were placed in serum-reduced culture medium for 12 hours and then treated as stated for 4 hours. After treatment, slides were placed in a parallel plate flow chamber (GlycoTech; Gaithersburg, MD), as described previously^11^. Briefly, peripheral blood mononuclear cells (PBMC; Sanguine Biosciences; Valencia, CA) were resuspended in Hank’s Buffered Salt Solution (HBSS) at a concentration of 5 × 10^5 ^cells/ml. Cells were then flowed over treated monolayers at a shear stress of 1 dyn/cm^2^ for 7 minutes, and non-adherent cells were then removed with HBSS at 2 dyn/cm^2^ for 2 minutes. Eight fields were randomly captured and adhered leukocytes were counted by a masked observer. Data are shown as the average of the eight captured fields for one slide and reported as adherent cells per mm^2^.

### NFkB Activity Assay

HRMEC were seeded on 96-well black-walled, clear bottom plates. Each well was transfected, as previously described^81^, with 200 ng NFkB-responsive luciferase constructs from the Cignal NFkB Reporter Assay (Qiagen). Cells were treated with vehicle or TNFα (1 ng/ml) in the presence or absence of 11,12-EET (0.5 μM) or 19,20-EDP (0.5 μM) with AUDA (10 μ M) for 24 hours after initiating transfection. Luciferase was quantified using a Dual-Glo Luciferase Assay System (Promega; Madison, WI) and data are reported as the relative ratio of firefly-to-Renilla luciferase.

### Retinal Leukostasis

All experiments were approved by the Vanderbilt University Institutional Animal Care and Use Committee and were performed in accordance with the ARVO Statement for the Use of Animals in Ophthalmic and Vision Research. Six-week old C57BL/6J mice (Charles Rivers; Wilmington, MA) were injected intravitreally with 2 μl of vehicle (0.1% DMSO and 0.3% EtOH), TNFα (50 ng/ml), TNFα with 11,12-EET (0.5 μM) plus AUDA (10 μM), or TNFα with 19,20-EDP (0.5 μM) plus AUDA (10 μM). As described previously^11^, 6 hours after treatment mice were anesthetized with ketamine and xylazine and perfused with 0.9% saline for 1 minute, followed by FITC-conjugated concanavalin-A (40 μg/ml in 2.5 ml PBS; Vector Laboratories; Burlingame, CA). Mice were then perfused with saline for 5 minutes to remove any non-adherent leukocytes. Retinas were immediately dissected into 4% paraformaldehyde, flat-mounted, and images were captured with an AX70 upright microscope (Olympus; Tokyo, Japan) and DP71 digital camera (Olympus) at 4x magnification. Adherent leukocytes in the vasculature were counted and divided by the total retinal vascular area.

### Statistical Analysis

Data were analyzed with the Prism software (GraphPad; La Jolla, CA) using analysis of variance with Fisher’s LSD post hoc analysis. Values of p < 0.05 were considered statistically significant.

## Additional Information

**How to cite this article**: Capozzi, M. E. *et al*. Epoxygenated Fatty Acids Inhibit Retinal Vascular Inflammation. *Sci. Rep.* **6**, 39211; doi: 10.1038/srep39211 (2016).

**Publisher’s note:** Springer Nature remains neutral with regard to jurisdictional claims in published maps and institutional affiliations.

## Supplementary Material

Supplementary Figures
