# Supplementary material for: Epoxygenated Fatty Acids Inhibit Retinal Vascular Inflammation
Source: Sci Rep. 2016 Dec 14;6:39211. doi: 10.1038/srep39211 (PMC5155241; doi:10.1038/srep39211)
Supplement: Supplementary Figures [file srep39211-s1.pdf]

Epoxygenated Fatty Acids Inhibit Retinal Vascular Inflammation

Megan E. Capozzi[1], Sandra S. Hammer[2], Gary W. McCollum[2], John S. Penn[1,2,*]

[1]Vanderbilt University, Department of Molecular Physiology and Biophysics, Nashville, TN, USA.

[2]Vanderbilt University Medical Center, Department of Ophthalmology and Visual Sciences, Nashville, TN, USA.

*Correspondence and requests for materials should be addressed to john.penn@vanderbilt.edu

Supplementary Information

Supplementary Figure S1

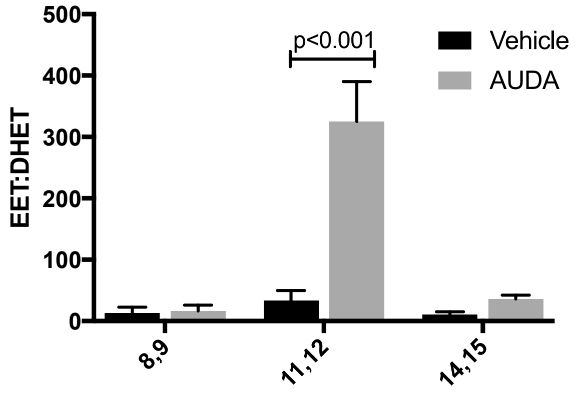

Supplementary Figure S1. AUDA alters EET-to-DHET ratios. To verify inhibition of epoxide hydrolysis by AUDA, EET:DHET ratio was measured in AUDA-treated HRMEC. EET:DHET for each isomer was increased, but only the ratio of the 11,12 regioisomer was significantly increased. Each bar represents mean ± SD (n=3).

Supplementary Figure S2

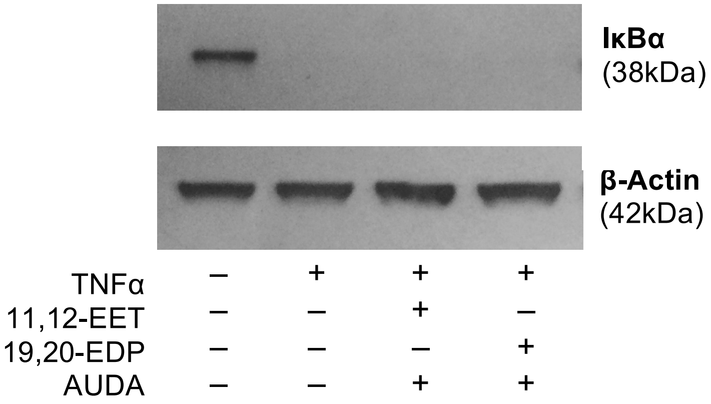

Supplementary Figure S2. 11,12-EET and 19,20-EDP do not directly exert their effect on IκBα in retinal microvascular endothelial cells. Treatment with 11,12-EET or 19,20-EDP with AUDA inhibition, as was used in the NFκB activity assay (**Figure 5**), did not prevent TNFα-induced IκBα degradation, suggesting an indirect mechanism of epoxygenated fatty acid inhibition of NFκB in HRMEC.
